# Crystal structure and Hirshfeld surface analysis of 2-chloro-*N*-(4-meth­oxy­phen­yl)acetamide

**DOI:** 10.1107/S205698902200576X

**Published:** 2022-06-07

**Authors:** Mohcine Missioui, Walid Guerrab, Intissar Nchioua, Abderrazzak El Moutaouakil Ala Allah, Camille Kalonji Mubengayi, Abdulsalam Alsubari, Joel T. Mague, Youssef Ramli

**Affiliations:** a Laboratory of Medicinal Chemistry, Drug Sciences Research Center, Faculty of Medicine and Pharmacy, Mohammed V University in Rabat, Morocco; b Laboratoire de Chimie et Biochimie, Institut Superieur des Techniques Medicales de Kinshasa, Republique Democratique du , Congo; c Laboratory of Medicinal Chemistry, Faculty of Clinical Pharmacy, 21 September University, Yemen; d Department of Chemistry, Tulane University, New Orleans, LA 70118, USA

**Keywords:** crystal structure, hydrogen bond, C—H⋯π(ring) inter­action, acetamide, Hirshfeld surface

## Abstract

In the title mol­ecule, C_9_H_10_ClNO_2_, the meth­oxy group lies very close to the plane of the phenyl ring while the acetamido group is twisted out of this plane by 28.87 (5)°. In the crystal, a three-dimensional structure is generated by N—H⋯O, C—H⋯O and C—H⋯Cl hydrogen bonds plus C—H⋯π(ring) inter­actions. A Hirshfeld surface analysis of the inter­molecular inter­actions was performed and indicated that C⋯H/H⋯C inter­actions make the largest contribution to the surface area (33.4%).

## Abstract 2

The meth­oxy group lies very close to the plane of the phenyl ring while the acetamido group is twisted out of this plane. In the crystal, N—H⋯O and C—H⋯O hydrogen bonds form layers of mol­ecules parallel to the *ab* plane. The layers are connected by C—H⋯Cl hydrogen bonds and C—H⋯π(ring) inter­actions, forming a three-dimensional structure.

## 1. Chemical context

Amides play a very important role in organic synthesis, including the production of medicines, functional materials, and bioactive mol­ecules (Alcaide *et al.*, 2007 ▸; Zhang *et al.*, 2012 ▸; García-Álvarez *et al.*, 2013 ▸; Ramli & Essassi, 2015 ▸; Álvarez-Pérez *et al.*, 2019 ▸). In particular, *N*-aryl­acetamides are significant inter­mediates for the synthesis of medicinal, agrochemical, and pharmaceutical compounds (Beccalli *et al.*, 2007 ▸; Valeur & Bradley, 2009 ▸; Allen & Williams, 2011 ▸; Missioui *et al.*, 2021 ▸, 2022*a* ▸,*b* ▸,*c* ▸). Given the wide range of therapeutic applications for such compounds, and in a continuation of our research efforts to synthesize more *N*-aryl­acetamides (Missioui *et al.*, 2020 ▸; Guerrab *et al.*, 2021 ▸), we report the synthesis, mol­ecular and crystal structure and Hirshfeld surface analysis of the title compound, 2-chloro-*N*-(4-meth­oxy­phen­yl)acetamide. 
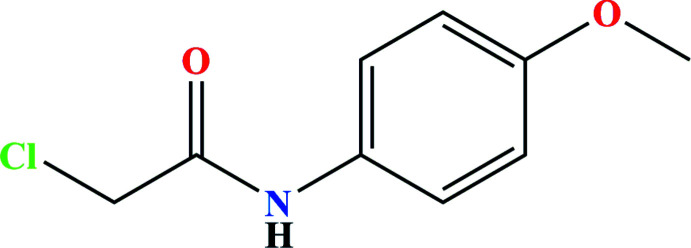


## 2. Structural commentary

**Figure 1:**
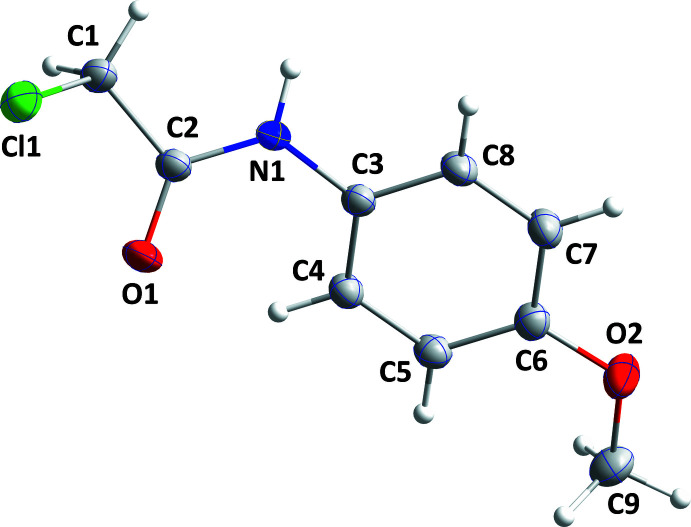
The mol­ecular structure of the title mol­ecule with labelling scheme and 50% probability ellipsoids.

The meth­oxy group lies close to the mean plane of the phenyl ring C3–C8, as indicated by the C7—C6—O2—C9 torsion angle of −174.61 (10)° and atom C9 deviating by only 0.065 (1) Å from the mean plane through the C3–C8 ring. In contrast, the acetamido group is rotated out of the above plane with the dihedral angle between the mean plane through the C3–C8 ring and that defined by N1/C2/C1/O1 being 28.87 (5)° (Fig. 1 ▸). The sum of the angles about N1 is 360.0 (9)°, indicating it to be planar (*sp* ^2^ hybridization). The Cl1—C1—C2—O1 torsion angle is 52.89 (12)°, illustrating a + *synclinal* (+ *gauche*) conformation about the C1—C2 bond. This places atom Cl1 at 1.299 (1) Å from the plane defined by C1, C2, N1 and O1.

## 3. Supra­molecular features

**Figure 2:**
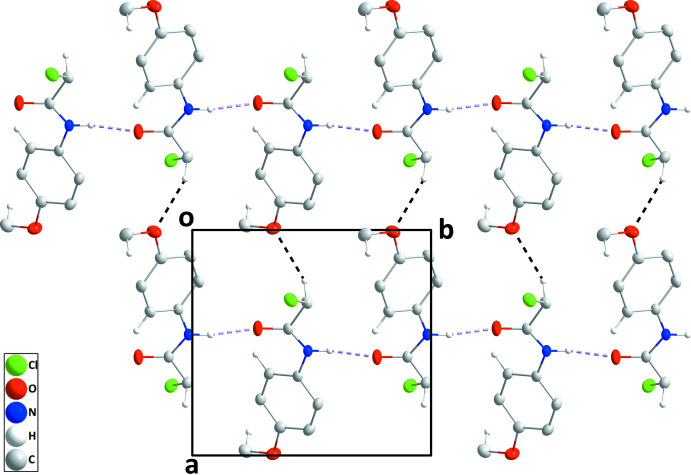
A portion of one layer of the crystal packing viewed along the *c*-axis direction with N—H⋯O and C—H⋯O hydrogen bonds depicted, respectively, by violet and black dashed lines. Non-inter­acting hydrogen atoms are omitted for clarity.

**Figure 3:**
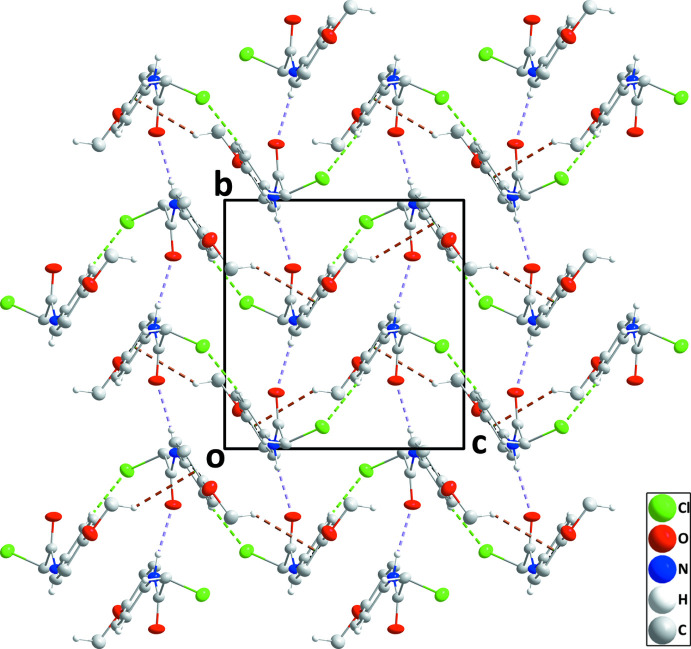
Packing viewed along the *a*-axis direction with N—H⋯O, C—H⋯O and C—H⋯Cl hydrogen bonds depicted, respectively by violet, black and light green dashed lines. C—H⋯π(ring) inter­actions are depicted by brown dashed lines and non-inter­acting hydrogen atoms are omitted for clarity.

In the crystal, N1—H1⋯O1 hydrogen bonds (Table 1 ▸) form helical chains along the 2_1_ axes. These chains are linked by C1—H1*A*⋯O2 hydrogen bonds (Table 1 ▸), forming layers of mol­ecules parallel to the *ab* plane (Fig. 2 ▸). The layers are linked by weak C4—H4⋯Cl1 hydrogen bonds as well as by C9—H9*B*⋯*Cg*1 inter­actions (Table 1 ▸) to generate the final three-dimensional structure (Fig. 3 ▸). As the shortest distance between parallel phenyl rings is 5.1075 (7) Å, there are no π–π stacking inter­actions present.

## 4. Database survey

**Figure 4:**
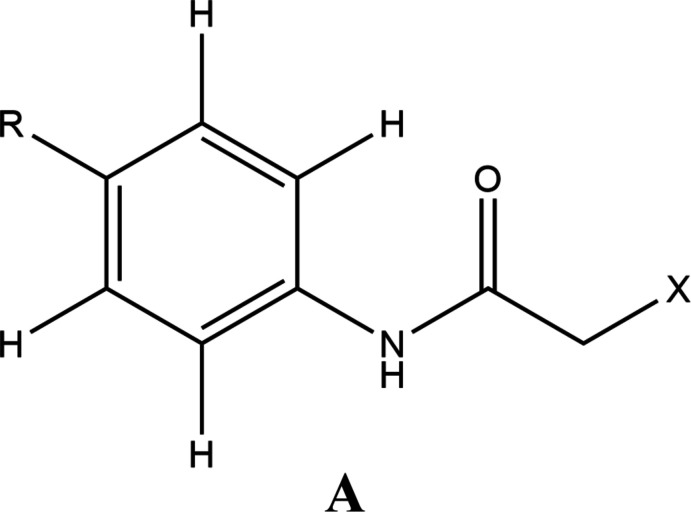
Fragment **A** used in the Cambridge Structural Database search.

A search of the Cambridge Structural Database (CSD, updated to March 2022; Groom *et al.*, 2016 ▸) using the fragment **A** (Fig. 4 ▸, *R* = undefined, *X* = halogen) yielded 15 hits of which 13 had *X* = Cl and *R* = OEt (DELZIE; Zhang *et al.*, 2006 ▸), COOEt (HEGLOW; Behbehani & Ibrahim, 2012 ▸), F (JODQEZ; Kang *et al.*, 2008 ▸), S(O)_2_NH(C_3_HNO(CH_3_)) (NULZEC; Murtaza *et al.*, 2019 ▸), SO_2_NH_2_ (PINXAO; Florke & Saeed, 2018 ▸; QUYRIM; Akkurt *et al.*, 2010 ▸), SMe (QUGTEU; Mongkholkeaw *et al.*, 2020 ▸), H (RIYWIG; Gowda *et al.*, 2008 ▸), NO_2_ (WEPGEE; Wen *et al.*, 2006 ▸; WEPGEE01; Gowda *et al.*, 2007*a* ▸), Cl (WINSUI; Gowda *et al.*, 2007*b* ▸), MeC(=O) (XABWEF; Ashraf *et al.*, 2016 ▸) and Me (XICMAY; Gowda *et al.*, 2007*c* ▸). The last two hits had *X* = Br and *R* = Br (FOWYIA; Gowda *et al.*, 2009 ▸) and CH_2_CH_2_O_2_CC(F)(SPh)(NO_2_) (VAGCOV; Takeuchi *et al.*, 1988 ▸). In general, the conformation of the haloacetamide portion is quite similar in all structures, as is the formation of infinite chains by N—H⋯O hydrogen bonds and these are comparable to the features found in the title structure. In DELZIE and XABWEF, C—H⋯π(ring) inter­actions assist in the packing, as also observed for the title mol­ecule.

## 5. Hirshfeld surface analysis

**Figure 5:**
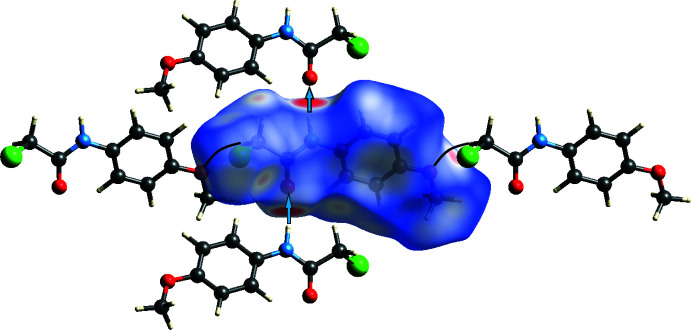
The Hirshfeld surface of the title mol­ecule with two adjacent mol­ecules involved in the N—H⋯O, hydrogen bonded chain and two involving the C1—H1*A*⋯O2 hydrogen bonds. The former inter­action is depicted by blue arrows and the latter by curved black lines.

**Figure 6:**
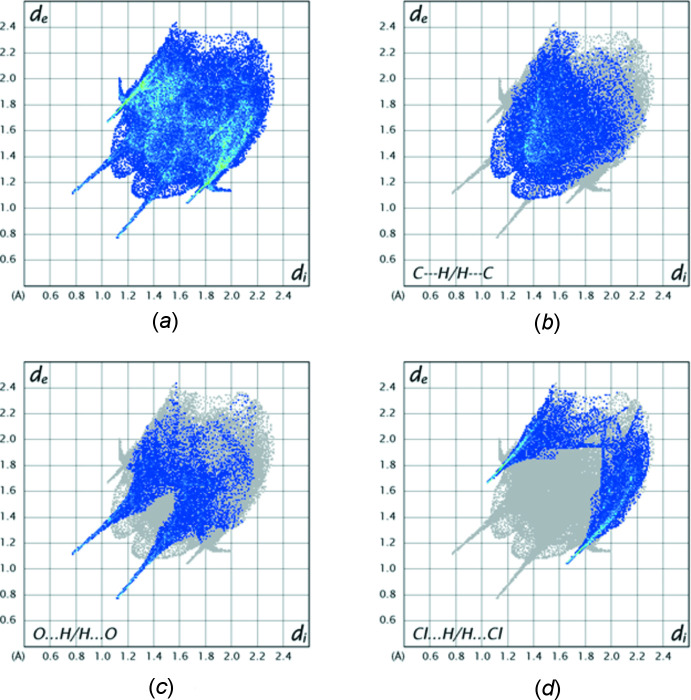
Fingerprint plots for the title mol­ecule: (*a*), all inter­molecular inter­actions; (*b*), C⋯H/H⋯C inter­actions; (*c*), O⋯H/H⋯O inter­actions; (*d*), Cl⋯H/H⋯Cl inter­actions.

The analysis was performed with *CrystalExplorer 21.5* (Spackman *et al.*, 2021 ▸) with the details of the pictorial output described in a recent publication (Tan *et al.*, 2019 ▸). Fig. 5 ▸ shows the *d*_norm_ surface for the asymmetric unit plotted over the range −0.5547 to 0.9665 arbitrary units together with two adjacent mol­ecules that are part of one infinite chain and two in adjacent chains (*cf*. Fig. 2 ▸). The bright-red spots at the top and bottom indicate the N—H⋯O hydrogen bonds (blue arrows) while the fa­inter ones at the far right and left indicate the C— H⋯O hydrogen bonds linking the chains (curved black lines) while that below and to the right of the Cl atom represents the weak C—H⋯Cl hydrogen bonds. Fig. 6 ▸ *a* is the fingerprint plot showing all inter­molecular inter­actions while Fig. 6 ▸ *b*–6*d* show these resolved into C⋯H/H⋯C (33.4%), O⋯H/H⋯O (19.5%) and Cl⋯H/H⋯Cl (20%) inter­actions, respectively.

## 6. Synthesis and crystallization

0.047 mol of 4-methoxyaniline were dissolved in 40 mL of pure acetic acid and put in an ice bath. Subsequently, chloro­acetyl chloride (0.047 mol) was added portionwise under stirring. At the end of the reaction, a solution of sodium acetate (35 mL) was added and a solid precipitate appeared after 30 min of stirring at room temperature. The resulting solid was filtered and washed with cold water, dried and recrystallized from ethanol to give the title compound as colourless crystals.

Yield 80%, m.p. = 398.6–400.3 K, FT–IR (ATR, υ, cm^−1^) 3292 (υ N—H amide), 1029 (υ N—C amide), 1660 (υ C=O amide), 3073 (υ C—H_arom_), 827 (υ C—Cl), 2959 (υ C—H,CH_2_), ^1^H NMR (DMSO–*d*_6_) δ pm: 3.74 (3H, *s*, CH_3_); 4.24 (2H, *s*, CH_2_), 6.93–7.5 (4H, *m*, *J* = 1.3 Hz, H_arom_), 10.23 (1H, *s*, NH), ^13^C NMR (DMSO–*d*_6_) δ ppm: 43.48 (CH_2_), 55.23 (CH_3_), 131.53 (C_arom_—N), 155.51 (C_arom_—O), 113.92–120.92 (C_arom_), 164.13 (C=O); HRMS (ESI–MS) (*m*/*z*) calculated for C_9_H_10_ClNO_2_ 199.04, found 199.0105.

## 7. Refinement

Crystal data, data collection and structure refinement details are summarized in Table 2 ▸. Hydrogen atoms attached to carbon were placed in idealized positions and included as riding contributions with isotropic displacement parameters fixed at 1.2*U*_eq_(C) (1.5 for the methyl group). The N-bound H atom was found in a difference-Fourier map and refined with a DFIX 0.91 0.01 instruction and an independent isotropic displacement parameter.

## Supplementary Material

Crystal structure: contains datablock(s) global, I. DOI: 10.1107/S205698902200576X/vm2264sup1.cif

Structure factors: contains datablock(s) I. DOI: 10.1107/S205698902200576X/vm2264Isup2.hkl

Supporting information file. DOI: 10.1107/S205698902200576X/vm2264Isup3.cml

CCDC reference: 2175514

Additional supporting information:  crystallographic information; 3D view; checkCIF report

## Figures and Tables

**Table 1:** Hydrogen-bond geometry (Å, °) *Cg*1 is the centroid of the C3–C8 benzene ring.

*D*—H⋯*A*	*D*—H	H⋯*A*	*D*⋯*A*	*D*—H⋯*A*
N1—H1⋯O1^i^	0.89 (1)	2.01 (1)	2.8910 (11)	171 (1)
C1—H1*A*⋯O2^ii^	0.99	2.48	3.3347 (13)	145
C4—H4⋯Cl1^iii^	0.95	2.83	3.7646 (10)	167
C9—H9*B*⋯*Cg*1^iv^	0.98	2.72	3.5020 (13)	137

Symmetry codes: (i) 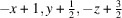 ; (ii) 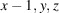 ; (iii) 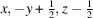 ; (iv) 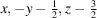 .

**Table 2:** Experimental details

Crystal data	
Chemical formula	C_9_H_10_ClNO_2_
*M*_r_	199.63
Crystal system, space group	Monoclinic, *P*2_1_/*c*
Temperature (K)	172
*a*, *b*, *c* (Å)	10.0939 (5), 9.6423 (5), 10.2799 (5)
β (°)	115.531 (2)
*V* (Å^3^)	902.83 (8)
*Z*	4
Radiation type	Mo *K*α
μ (mm^−1^)	0.39
Crystal size (mm)	0.29 × 0.25 × 0.09

Data collection	
Diffractometer	Bruker D8 QUEST PHOTON 3 diffractometer
Absorption correction	Numerical (*SADABS*; Krause *et al.*, 2015 ▸)
*T*_min_, *T*_max_	0.91, 0.97
No. of measured, independent and observed [*I* > 2σ(*I*)] reflections	43210, 2871, 2508
*R*_int_	0.034
(sin θ/λ)_max_ (Å^−1^)	0.726

Refinement	
*R*[*F* ^2^ > 2σ(*F* ^2^)], *wR*(*F* ^2^), *S*	0.032, 0.091, 1.10
No. of reflections	2871
No. of parameters	123
No. of restraints	1
H-atom treatment	H atoms treated by a mixture of independent and constrained refinement
Δρ_max_, Δρ_min_ (e Å^−3^)	0.38, −0.21

Computer programs: *APEX3* and *SAINT* (Bruker, 2020 ▸), *SHELXT* (Sheldrick, 2015*a* ▸), *SHELXL2018/1* (Sheldrick, 2015*b* ▸), *DIAMOND* (Brandenburg & Putz, 2012 ▸) and *SHELXTL* (Sheldrick, 2008 ▸).

## supplementary crystallographic information

### Crystal data

**Table:**

C_9_H_10_ClNO_2_	*F*(000) = 416
*M**_r_* = 199.63	*D*_x_ = 1.469 Mg m^−^^3^
Monoclinic, *P*2_1_/*c*	Mo *K*α radiation, λ = 0.71073 Å
*a* = 10.0939 (5) Å	Cell parameters from 9903 reflections
*b* = 9.6423 (5) Å	θ = 2.2–31.1°
*c* = 10.2799 (5) Å	µ = 0.39 mm^−^^1^
β = 115.531 (2)°	*T* = 172 K
*V* = 902.83 (8) Å^3^	Plate, colourless
*Z* = 4	0.29 × 0.25 × 0.09 mm

### Data collection

**Table:**

Bruker D8 QUEST PHOTON 3 diffractometer	2871 independent reflections
Radiation source: fine-focus sealed tube	2508 reflections with *I* > 2σ(*I*)
Graphite monochromator	*R*_int_ = 0.034
Detector resolution: 7.3910 pixels mm^-1^	θ_max_ = 31.1°, θ_min_ = 3.1°
φ and ω scans	*h* = −14→14
Absorption correction: numerical (*SADABS*; Krause *et al.*, 2015)	*k* = −13→13
*T*_min_ = 0.91, *T*_max_ = 0.97	*l* = −14→14
43210 measured reflections	

### Refinement

**Table:**

Refinement on *F*^2^	Primary atom site location: dual
Least-squares matrix: full	Secondary atom site location: difference Fourier map
*R*[*F*^2^ > 2σ(*F*^2^)] = 0.032	Hydrogen site location: mixed
*wR*(*F*^2^) = 0.091	H atoms treated by a mixture of independent and constrained refinement
*S* = 1.10	*w* = 1/[σ^2^(*F*_o_^2^) + (0.0462*P*)^2^ + 0.2203*P*] where *P* = (*F*_o_^2^ + 2*F*_c_^2^)/3
2871 reflections	(Δ/σ)_max_ = 0.001
123 parameters	Δρ_max_ = 0.38 e Å^−^^3^
1 restraint	Δρ_min_ = −0.21 e Å^−^^3^

### Special details

**Table:**

Experimental. The diffraction data were obtained from 7 sets of frames, each of width 0.5° in ω or φ, collected with scan parameters determined by the "strategy" routine in *APEX3*. The scan time was 6 sec/frame.
Geometry. All esds (except the esd in the dihedral angle between two l.s. planes) are estimated using the full covariance matrix. The cell esds are taken into account individually in the estimation of esds in distances, angles and torsion angles; correlations between esds in cell parameters are only used when they are defined by crystal symmetry. An approximate (isotropic) treatment of cell esds is used for estimating esds involving l.s. planes.
Refinement. Refinement of F^2^ against ALL reflections. The weighted R-factor wR and goodness of fit S are based on F^2^, conventional R-factors R are based on F, with F set to zero for negative F^2^. The threshold expression of F^2^ > 2sigma(F^2^) is used only for calculating R-factors(gt) etc. and is not relevant to the choice of reflections for refinement. R-factors based on F^2^ are statistically about twice as large as those based on F, and R- factors based on ALL data will be even larger. H-atoms attached to carbon were placed in calculated positions (C—H = 0.95 - 0.99 Å) and were included as riding contributions with isotropic displacement parameters 1.2 - 1.5 times those of the attached atoms. That attached to nitrogen was placed in a location derived from a difference map and refined with a DFIX 0.91 0.01 instruction.

### Fractional atomic coordinates and isotropic or equivalent isotropic displacement parameters (Å^2^)

**Table:**

	*x*	*y*	*z*	*U*_iso_*/*U*_eq_	
Cl1	0.30989 (3)	0.41222 (3)	0.90827 (3)	0.03169 (9)	
O1	0.43630 (10)	0.27410 (8)	0.71560 (10)	0.03148 (19)	
O2	0.99240 (9)	0.34016 (9)	0.56309 (9)	0.03316 (19)	
N1	0.53618 (9)	0.48405 (8)	0.70583 (9)	0.02160 (17)	
H1	0.5353 (17)	0.5749 (9)	0.7222 (16)	0.034 (4)*	
C1	0.32272 (11)	0.47958 (10)	0.75260 (11)	0.02423 (19)	
H1A	0.226444	0.470886	0.667909	0.029*	
H1B	0.348725	0.579198	0.767150	0.029*	
C2	0.43834 (11)	0.40146 (10)	0.72459 (10)	0.02147 (19)	
C3	0.64850 (11)	0.44121 (10)	0.66649 (10)	0.02036 (18)	
C4	0.63724 (11)	0.32251 (10)	0.58566 (11)	0.02264 (19)	
H4	0.552126	0.265906	0.555512	0.027*	
C5	0.75029 (11)	0.28609 (10)	0.54860 (10)	0.02332 (19)	
H5	0.742024	0.204773	0.493437	0.028*	
C6	0.87484 (11)	0.36845 (11)	0.59218 (11)	0.02389 (19)	
C7	0.88452 (12)	0.48995 (11)	0.66987 (12)	0.0275 (2)	
H7	0.968462	0.547843	0.697712	0.033*	
C8	0.77218 (12)	0.52620 (10)	0.70637 (11)	0.0249 (2)	
H8	0.779097	0.609174	0.758744	0.030*	
C9	0.97987 (13)	0.22369 (13)	0.47330 (13)	0.0325 (2)	
H9A	1.070019	0.214361	0.460154	0.049*	
H9B	0.896160	0.237045	0.379304	0.049*	
H9C	0.964803	0.139514	0.518658	0.049*	

### Atomic displacement parameters (Å^2^)

**Table:**

	*U*^11^	*U*^22^	*U*^33^	*U*^12^	*U*^13^	*U*^23^
Cl1	0.03574 (16)	0.03273 (15)	0.03447 (15)	0.00576 (10)	0.02258 (12)	0.00686 (10)
O1	0.0429 (5)	0.0163 (3)	0.0466 (5)	−0.0005 (3)	0.0300 (4)	0.0011 (3)
O2	0.0278 (4)	0.0390 (5)	0.0389 (4)	−0.0049 (3)	0.0202 (3)	−0.0107 (3)
N1	0.0247 (4)	0.0154 (4)	0.0269 (4)	−0.0001 (3)	0.0133 (3)	−0.0006 (3)
C1	0.0259 (5)	0.0214 (4)	0.0278 (5)	0.0031 (3)	0.0139 (4)	0.0043 (4)
C2	0.0253 (4)	0.0181 (4)	0.0226 (4)	0.0010 (3)	0.0118 (4)	0.0022 (3)
C3	0.0228 (4)	0.0177 (4)	0.0213 (4)	0.0002 (3)	0.0102 (3)	0.0013 (3)
C4	0.0239 (4)	0.0204 (4)	0.0240 (4)	−0.0033 (3)	0.0107 (3)	−0.0022 (3)
C5	0.0275 (5)	0.0206 (4)	0.0232 (4)	−0.0013 (3)	0.0121 (4)	−0.0027 (3)
C6	0.0252 (4)	0.0255 (5)	0.0229 (4)	−0.0010 (4)	0.0122 (4)	−0.0002 (3)
C7	0.0278 (5)	0.0256 (5)	0.0320 (5)	−0.0071 (4)	0.0156 (4)	−0.0051 (4)
C8	0.0286 (5)	0.0194 (4)	0.0283 (5)	−0.0041 (4)	0.0139 (4)	−0.0042 (3)
C9	0.0336 (6)	0.0345 (6)	0.0336 (6)	0.0051 (4)	0.0184 (5)	−0.0027 (4)

### Geometric parameters (Å, º)

**Table:**

Cl1—C1	1.7828 (10)	C4—C5	1.3947 (14)
O1—C2	1.2310 (12)	C4—H4	0.9500
O2—C6	1.3704 (13)	C5—C6	1.3880 (14)
O2—C9	1.4243 (14)	C5—H5	0.9500
N1—C2	1.3457 (13)	C6—C7	1.3973 (15)
N1—C3	1.4194 (13)	C7—C8	1.3840 (15)
N1—H1	0.893 (9)	C7—H7	0.9500
C1—C2	1.5172 (14)	C8—H8	0.9500
C1—H1A	0.9900	C9—H9A	0.9800
C1—H1B	0.9900	C9—H9B	0.9800
C3—C4	1.3900 (13)	C9—H9C	0.9800
C3—C8	1.3989 (14)		
			
C6—O2—C9	117.06 (9)	C6—C5—C4	120.11 (9)
C2—N1—C3	126.46 (8)	C6—C5—H5	119.9
C2—N1—H1	119.0 (10)	C4—C5—H5	119.9
C3—N1—H1	114.5 (10)	O2—C6—C5	124.39 (9)
C2—C1—Cl1	110.42 (7)	O2—C6—C7	115.96 (9)
C2—C1—H1A	109.6	C5—C6—C7	119.65 (9)
Cl1—C1—H1A	109.6	C8—C7—C6	120.20 (9)
C2—C1—H1B	109.6	C8—C7—H7	119.9
Cl1—C1—H1B	109.6	C6—C7—H7	119.9
H1A—C1—H1B	108.1	C7—C8—C3	120.30 (9)
O1—C2—N1	124.69 (9)	C7—C8—H8	119.8
O1—C2—C1	121.35 (9)	C3—C8—H8	119.8
N1—C2—C1	113.91 (8)	O2—C9—H9A	109.5
C4—C3—C8	119.36 (9)	O2—C9—H9B	109.5
C4—C3—N1	122.71 (9)	H9A—C9—H9B	109.5
C8—C3—N1	117.88 (9)	O2—C9—H9C	109.5
C3—C4—C5	120.33 (9)	H9A—C9—H9C	109.5
C3—C4—H4	119.8	H9B—C9—H9C	109.5
C5—C4—H4	119.8		
			
C3—N1—C2—O1	3.07 (17)	C9—O2—C6—C5	4.72 (16)
C3—N1—C2—C1	−174.47 (9)	C9—O2—C6—C7	−174.61 (10)
Cl1—C1—C2—O1	52.89 (12)	C4—C5—C6—O2	178.93 (10)
Cl1—C1—C2—N1	−129.47 (8)	C4—C5—C6—C7	−1.77 (15)
C2—N1—C3—C4	27.78 (15)	O2—C6—C7—C8	−179.00 (10)
C2—N1—C3—C8	−154.97 (10)	C5—C6—C7—C8	1.64 (16)
C8—C3—C4—C5	2.04 (15)	C6—C7—C8—C3	0.34 (16)
N1—C3—C4—C5	179.25 (9)	C4—C3—C8—C7	−2.17 (15)
C3—C4—C5—C6	−0.08 (15)	N1—C3—C8—C7	−179.52 (9)

### Hydrogen-bond geometry (Å, º)

*Cg*1 is the centroid of the C3–C8 benzene ring.

**Table:**

*D*—H···*A*	*D*—H	H···*A*	*D*···*A*	*D*—H···*A*
N1—H1···O1^i^	0.89 (1)	2.01 (1)	2.8910 (11)	171 (1)
C1—H1*A*···O2^ii^	0.99	2.48	3.3347 (13)	145
C4—H4···Cl1^iii^	0.95	2.83	3.7646 (10)	167
C9—H9*B*···*Cg*1^iv^	0.98	2.72	3.5020 (13)	137

Symmetry codes: (i) −*x*+1, *y*+1/2, −*z*+3/2; (ii) *x*−1, *y*, *z*; (iii) *x*, −*y*+1/2, *z*−1/2; (iv) *x*, −*y*−1/2, *z*−3/2.
